# Investigations of 2D PtS_2_’s Saturable Absorption Characteristic and Its Optimization to OPO’s Operation

**DOI:** 10.3390/nano12101670

**Published:** 2022-05-13

**Authors:** Xinyu Hu, Jing Wang, Heze Guo, Kai Jiang, Wenjing Tang, Wei Xia

**Affiliations:** School of Physics and Technology, University of Jinan, Jinan 250022, China; hxy2237623598@163.com (X.H.); ghz772767365@163.com (H.G.); sps_jiangk@ujn.edu.cn (K.J.); sps_xiaw@ujn.edu.cn (W.X.)

**Keywords:** nanotechnology, 2D PtS_2_, saturable absorption, nonlinear frequency conversion, optical parametric oscillator

## Abstract

A 6.2 nm-thickness platinum disulfide (PtS_2_) film was prepared by electron beam evaporation with post vulcanization. The nonlinear transmittance was measured by power scanning method and the modulation depth is fitted to be 13%. Based on the transmittance curve, saturable absorption parameters of PtS_2_ are calculated with inhomogeneously broadening mechanism, including 6.4298 × 10^−19^ cm^−2^ ground-state absorption cross-section, 2.5927 × 10^−19^ cm^−2^ excited-state absorption cross-section, and 1.043 ms excited-state lifetime. The PtS_2_ film combined with active time management was implemented to modulate the fundamental light of optical parametric oscillator (OPO). Owing to the nonlinear absorption property of PtS_2_, the operation of Q-switched OPO was optimized in both the experiment and dynamical theory. In particular, the conversion efficiency was experimentally improved by 13.2%. The pump-to-signal conversion efficiency went up to 3.29%, which is the highest conversion value reported so far. The theoretical values fit the experiment well, which are from the Gaussian rate equations with PtS_2_’s saturable-absorption characteristic.

## 1. Introduction

As passive modulators for laser pulses, two-dimensional (2D) transition metal sulfides (TMDCs) have attracted much attention in recent years, because of the advantages of fast recovery time, high modulation depth, and low preparing cost [1,2]. As a member of the TMDCs family, the few-layer platinum disulfide (PtS_2_) consists of S–Pt–S atoms in the plane, and there are strong covalent bonding and weak van der Waals bonding between the layers [3,4]. Few-layer PtS_2_ is promising in 1μm-and-above-wavelength laser modulation, and its bandgap ranges from 0.25 eV for bulk to 1.6 eV for monolayer [5,6,7,8,9]. At present, 2D PtS_2_ has been applied to fiber laser to obtain 1 μm or 1.5 μm-wavelength pulse [5,10,11]. Although 2D TMDC has been proven to have a high damage threshold and a high switching ratio, layered PtS_2_ has not been reported to be used in solid-state lasers as a passive modulator [12,13,14,15].

Based on second-order nonlinear frequency conversion, optical parametric oscillator (OPO) can generate 1.5–1.6 μm coherent eye-safe light [16,17]. Compared to extracavity OPO, the high photon density of fundamental laser for the intracavity OPO (IOPO) can be fully utilized to generate signal or idler pulses with high single-pulse energy. For a Q-switched IOPO, the shorter width and higher peak fundamental laser contribute to higher conversion [18,19]. Although the lasers Q-switched by 2D TMDCs are reported a lot, few of them have been applied to OPO’s fundamental lasers because of the low time-stability of pulse sequence [20,21,22,23]. If an active Q-switch is introduced to manage the action time of the saturable absorber (SA), the IOPO modulated by 2D PtS_2_ is prospected. Moreover, due to PtS_2_’s saturable absorption characteristic, the experimental optimization of Q-switched IOPO could be realized.

Dynamic rate equations are effective in guiding the experiments of lasers or OPOs based on PtS_2_ [24,25]. Compared with the plane-wave approximation results, the theoretical calculations are better fitted to the experimental results when considering the Gaussian spatial distribution of the fundamental photon [26,27]. So far, few works have been involved in the Gaussian rate equations of IOPOs. The saturation absorption parameters of PtS_2_ are needed to solve the dynamic equations, and they are also the keys to obtaining narrow-pulse and high peak-power lasers. As the energy band structure of PtS_2_ is still being researched, the values of SA parameters are rarely reported, including the ground-state absorption cross-section, the excited-state absorption cross-section, and the excited-state lifetime. Therefore, a dynamical investigation of lasers modulated by PtS_2_ has not been reported in the literature, especially not an IOPO pumped by it.

In this work, a few-layer PtS_2_ SA was prepared by the method of electron beam evaporation (EBE) method, and its optical properties were thoroughly investigated. Considering the inhomogeneously broadening mechanism, the SA parameters of PtS_2_ were estimated from the measured transmittance curves. Then, the dynamic coupling rate equations under the Gaussian spatial distribution were derived and solved for a Q-switched IOPO with PtS_2_. Finally, in our experiment, a signal-resonant KTP IOPO based on the prepared PtS_2_ SA was realized. The acousto-optic (AO), as an active modulator, was used to control the fundamental-pulse-repetition rate. The few-layer PtS_2_ SA was used to optimize the performance of Q-switched IOPO as a passive Q-switch. The optimization of PtS_2_ SA to IOPO was analyzed, including the pulse-width compression, the peak-power improvement, the pulse-sequence stability and the nonlinear-conversion-efficiency promotion. The theoretical results are in reasonable agreement with the experimental values.

## 2. Material Fabrication and Characterization in Experiment

**Figure 1:**
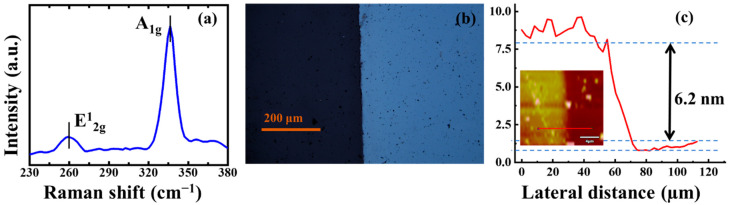
Characteristic of the prepared PtS_2_. (**a**) Optical micrograph; (**b**) Raman spectrum; (**c**) Atomic force microscopy.

PtS_2_ thin film was synthesized by metal film plus post-sulfurization treatment [28]. In the first step, the Pt film was deposited on c-cut sapphire (Al_2_O_3_ substrate) by using an electron beam evaporation furnace (ATS 500, HHV, West Sussex, UK). After that, the Pt-coated Al_2_O_3_ substrate was thermally annealed in a sulfur-rich ambiance for 30 min. During the thermal treatment, a flow of 80 sccm H_2_S was introduced for sulfurization and the Ar/H_2_ mixture of 225 sccm/75 sccm was employed as a carrier gas and reactive gas. Finally, the PtS_2_ sample was removed for characterization. The PtS_2_ SA is characteristically summarized in Figure 1. The Raman spectrum of the PtS_2_ SA was measured by a Raman spectrometer (Horiba LabRAM HR Evolution, Kyoto, Japan), excited by a 532 nm laser. As shown in Figure 1a, the two characteristic peaks at 260 cm^−1^ and 336 cm^−1^ correspond to the in-plane E^1^_2g_ vibrational mode and the out-of-plane A_1g_ vibrational mode, respectively. The frequency interval between the two peaks is 76 cm^−1^. As has already been established, the E^1^_2g_ mode is related to the in-plane vibration of the S and Pt atoms heading in opposite directions. The A_1g_ mode is related to the out-of-plane motion of the S atom, which reflects the coupling between layers. The A_1g_ peak of PtS_2_ prepared in this experiment is blue-shifted by 2 cm^−1^ compared with bulk-like PtS_2_, which implies the 2D nanostructure [12,14]. The optical micrograph (CX23, Olympus, Tokyo, Japan) measured in Figure 1b demonstrates the contrast between the PtS_2_ sample and pristine transparent sapphire substrate. The color contrast of the sample indicates that the sample is a few-layer film. Figure 1c shows the atomic force microscopy (AFM, Bruker Dimension Icon, Billerica, MA, USA) where the thickness and the corresponding height diagram are given. As illustrated in the figure, the thickness of PtS_2_ is approximately 6.2 nm. Considering the 5.03 Å thickness of single-layer and 3.07 Å interplanar spacing for PtS_2_ [29,30], the structure of the sample is approximately 8 layers, which corresponds to the abovementioned optical micrograph and Raman spectrum analysis results.

**Figure 2:**
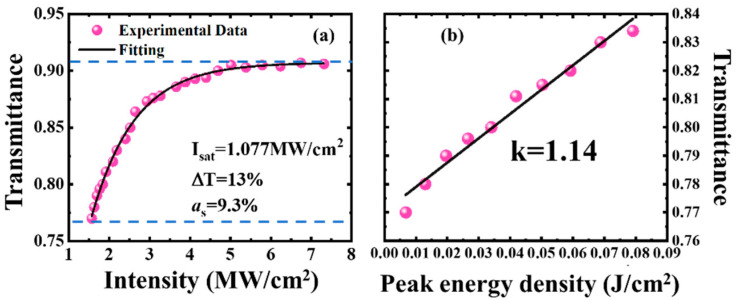
Optical characteristics of PtS_2_**.** (**a**) The transmittance of PtS_2_ SA versus incident power intensity. (**b**) The linear relation for low-power density.

The nonlinear transmittance of PtS_2_ SA versus different incident peak power intensity is measured by double-optical-path method [31]. An AO Q-switched solid-state laser (1.06 µm wavelength, 120 ns pulse width, 15 kHz repetition rate) was used as the laser source. The nonlinear transmittance of the sample is shown in Figure 2a. The approximated nonsaturable loss (α*_s_*), initial transmittance (*T*_0_), and saturation transient intensity (*I*_sat_) were fitted as 9.3%, 77.0%, and 1.077 MW/cm^2^, respectively, and then the modulation depth (Δ*T*) was calculated to be 13%. With the increasing peak-energy density at the low power density, the material transmittance increases linearly, as shown in Figure 2b. The slope obtained by linear fitting is 1.14.

## 3. Theoretical Evaluation of IOPO with 6.2 nm-Thick PtS_2_’s Saturable Absorption Characteristic

In theory, Q-switching dynamics is well explained by rate equations [32]. In this section, the Gaussian assumption and PtS_2_’s characteristic were considered for the dynamical model of a Q-switched Nd:YVO_4_/KTP IOPO with AO active time-management.

### 3.1. Gaussian Rate Equations of IOPO

The Gaussian transversal distribution and longitudinal distribution of the photon density in the cavity are considered in the rate equations, as well as the thermal effect of the gain medium. The average intracavity photon density ϕ(r,t) of the TEM_00_ mode can be represented by

(1) $$\phi \left(r,t\right)=\phi \left(0,t\right)\exp\left(-\frac{2r^{2}}{w_{l}^{2}}\right)$$

In this formula, *w_l_* is the average beam radius of the fundamental light.

(2) $$\phi_{j}\left(r,t\right)=\frac{w_{l}^{2}}{w_{j}^{2}}\phi_{j}\left(0,t\right)\exp\left(-\frac{2r^{2}}{w_{j}^{2}}\right)\left(j=g,a,y,p,s,i\right)$$

where $\phi_{j}(0,t)$(*j = s*, *i*) represents the intracavity photon densities of the signal light and idler light; $\phi_{j}(0,t)$(*j = g*, *a*, *y*) represents the fundamental light intracavity photon densities at the positions of Nd: YVO_4_ crystal, AO crystal, and PtS_2_ SA, respectively [33]; *w_j_* (*j = g*, *a*, *y*) represents the radius of the TEM_00_ mode in the abovementioned three positions. The relationship between the photon densities and the electric field is represented by $\phi (r,t)=\varepsilon E^{2}\left(r,t\right)/4\hbar \omega$. Considering the transverse Gaussian distribution of beam energy, *E_j_* (*r*, *t*) = *E_j_*(0, *t*)exp(-*r*^2^/*w_j_*^2^) can be derived, where *E*(*r*, *t*) is the electrical field. *E_j_* (0, *t*) (*j = p*, *s*, *i*) is the electrical field of the fundamental light, the signal light and the idler light on the laser axis. 

Under Gaussian assumption, the excited-state population densities $n_{y1}(r,t)$ of the saturable absorber can be expressed as

(3) $$n_{y1}\left(r,t\right)=n_{y1}\left(0,t\right)\exp\left(-\frac{2r^{2}}{w_{y}^{2}}\right)$$

The IOPO rate equation with the saturable absorption characteristic of PtS_2_ SA is obtained by referring to the previous research work [26,27,32,33].

(4) $$\begin{array}{l} \int_{0}^{\infty }\frac{d\phi \left(r,t\right)}{dt}\cdot 2\pi rdr=\\ \int_{0}^{\infty }\frac{1}{t_{r}}\cdot \left[2\sigma l_{g}n\left(r,t\right)\phi_{g}\left(r,t\right)-\left(\sigma_{g}-\sigma_{e}\right)l_{y}n_{y1}\left(r,t\right)\phi_{y}\left(r,t\right)-\sigma_{e}l_{y}n_{y0}\phi_{y}\left(r,t\right)\right]\\ \cdot 2\pi rdr-\int_{0}^{\infty }\frac{1}{t_{r}}\cdot \left[\delta_{T}\left(t\right)\phi_{g}\left(r,t\right)+\delta_{a}\left(t\right)\phi_{a}\left(r,t\right)\right]\cdot 2\pi rdr-\int_{0}^{\infty }\frac{\delta Iw_{k}^{2}}{8\hbar w_{l}^{2}}\cdot \frac{l_{opo}}{l}\cdot \frac{l_{KTP}}{l_{opo}}\\ \cdot E_{p}\left(r,t\right)E_{s}\left(r,t\right)E_{i}\left(r,t\right)\cdot 2\pi rdr \end{array}$$

(5) $$\int_{0}^{\infty }\frac{d\phi_{s}\left(r,t\right)}{dt}\cdot 2\pi rdr=\int_{0}^{\infty }\left[-\frac{\phi_{s}\left(r,t\right)}{\tau_{s}}+\frac{\delta I}{8\hbar }\cdot \frac{l_{KTP}}{l_{opo}}\cdot E_{p}\left(r,t\right)E_{s}\left(r,t\right)E_{i}\left(r,t\right)\right]\cdot 2\pi rdr$$

(6) $$\int_{0}^{\infty }\frac{d\phi_{i}\left(r,t\right)}{dt}\cdot 2\pi rdr=\int_{0}^{\infty }\left[-\frac{\phi_{i}\left(r,t\right)}{\tau_{s}}+\frac{\delta I}{8\hbar }\cdot \frac{l_{KTP}}{l_{opo}}\cdot E_{p}\left(r,t\right)E_{s}\left(r,t\right)E_{i}\left(r,t\right)\right]\cdot 2\pi rdr$$

(7) $$\int_{0}^{\infty }\frac{dn\left(r,t\right)}{dt}\cdot 2\pi rdr=\int_{0}^{\infty }\left[R_{in}e^{-\frac{2r^{2}}{{\bar{w}}_{p}^{2}}}-\sigma c\phi_{g}\left(r,t\right)n\left(r,t\right)-\frac{n\left(r,t\right)}{\tau }\right]\cdot 2\pi rdr$$

(8) $$\int_{0}^{r_{y}}\frac{dn_{y1}\left(r,t\right)}{dt}\cdot 2\pi rdr=\int_{0}^{r_{y}}\left[\frac{n_{y0}-n_{y1}\left(r,t\right)}{\tau_{y}}-\frac{\varepsilon_{p}}{4\hbar \omega_{p}}\cdot \sigma_{g}cn_{y1}\left(r,t\right)E_{p}^{2}\left(r,t\right)\right]\cdot 2\pi rdr$$

Equation (4) describes the loss of fundamental-light photons by thermal effect, AO crystal and nonlinear transformation of the KTP crystal. In the equation, $-\left[\left(\sigma_{g}-\sigma_{e}\right)l_{y}n_{y1}\left(r,t\right)\phi_{y}\left(r,t\right)+\sigma_{e}l_{y}n_{y0}\phi_{y}\left(r,t\right)\right]$ represents the loss of fundamental-light photons by PtS_2_ SA. The gain of signal-light and idler-light photons with time can be described by Equations (5) and (6). Equation (7) shows the loss of the population inversion in the laser medium. In the equations, *t_r_* is the round-trip time of the fundamental light. *l*, *l*_g_, *l_KTP_* and *l_OPO_* are the physical lengths of the laser cavity, the gain medium, KTP crystal, and OPO cavity. *ε_j_* (*j* = *p*, *s*, *i*) is the dielectric constant and the frequency of the three lights. In Equation (4), *δ_T_* is the diffractive loss caused by the gain medium’s thermal effect. *δ_a_* (*t*) can be written as [32],

(9) $$\delta_{a}\left(t\right)=\delta_{a}\exp\left[-{\left(\frac{t}{t_{AO}}\right)}^{2}\right]$$

where *t_AO_* and *δ_a_* are the turn-off time and the intrinsic diffraction loss of the AO Q-switch. *δ_a_* (*t*) is used to describe the loss function of the gradual AO modulator (AOM) caused by the exponential time delay. The basic parameters for calculating the rate equation refer to previous work [32,33,34,35].

### 3.2. PtS_2_ SA Parameters Derivation by Inhomogeneously Broadening Mechanism

For PtS_2_ SA, the change of the population inversion density is shown in Equation (8). *σ_g_* and *σ_e_* are the ground-state and excited-state absorption cross-sections of SA, which are essential parameters to characterize saturable-absorption properties of SA. By using the information in reference [33], we can obtain the relationship between them.

(10) $$\frac{\sigma_{g}}{\sigma_{e}}=\frac{\ln T_{0}}{\ln T_{\max}}$$

(11) $$\frac{\left(\sigma_{g}-\sigma_{e}\right)\times T_{0}}{hv}=k$$

The values of small-signal transmittance (*T*_0_) at a low power density, maximum transmittance (*T*_max_) at a high power density, and slope *k* were obtained, as shown in Figure 2. Then, the values of *σ_g_* and *σ_e_* for the 6.2 nm-thickness PtS_2_ SA can be estimated to be 6.4298 × 10^−19^ cm^−2^ and 2.5927 × 10^−19^ cm^−2^. Considering the inhomogeneously broadening mechanism, the excited-state lifetime of PtS_2_ is calculated to be 1.043 ms [33]. Table 1 demonstrates the calculated key parameters of PtS_2_ SA.

These parameters can be substituted into Equations (4)–(8), which are solved by computer programming. In addition, Equations (4), (7), and (8) could dynamically model a PtS_2_ SA passively Q-switched laser.

## 4. Optimized Experiment of Q-Switched IOPO Based on PtS_2_ SA

### 4.1. Experimental Setup

**Figure 3:**
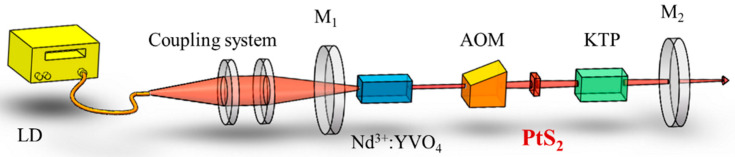
Experimental setup of the IOPO pumped by a doubly Q-switched Nd^3+^: YVO_4_ laser with AOM and PtS_2_ SA.

The experimental setup for doubly Q-switched Nd^3+^: YVO_4_ IOPO with AO modulator (AOM) and PtS_2_ SA is shown in Figure 3. A commercial LD with 400 µm beam diameter is used as the pump source, and the 808 nm-wavelength laser from LD is focused by the 1:0.5 coupled lens. The gain medium used in the experiment is a Nd:YVO_4_ laser crystal with a 3 × 3 × 5 mm^3^ size and 0.6 at.% Nd^3+^ dope. The two faces of the crystal were antireflection (AR)-coated at both 808 nm and 1064 nm wavelengths. The 5 × 5 × 20 mm^3^ KTP crystal with type II non-critical phase-matching configuration (θ = 90°, *φ* = 0°) along *X*-axis is applied to obtain a maximum effective nonlinear coefficient. The fundamental cavity length is about 125 mm, which is from mirror M_1_ to M_2_. The OPO cavity length is about 28 mm, and the signal light oscillates between M_2_ and the first side of KTP. The repetitions of AO were 15 kHz and 25 kHz, respectively.

A laser power meter (MAX500AD, Coherent Inc., Santa Clara, CA, USA) was employed to measure the laser average output power. Two fast photo-electronic diodes (with a rising time of 1 ns) and a digital oscilloscope (TDS620B, Tektronix, Beaverton, OR, USA) were used to measure the fundamental-pulse and the signal-pulse temporal characteristics of the output, respectively. A spectrometer (MS9710C, Anritsu, Atsugi, Japan), working in the wavelength range of 600–1750 nm, was employed to record the laser spectrum.

### 4.2. Experimental Results and Discussion

**Figure 4:**
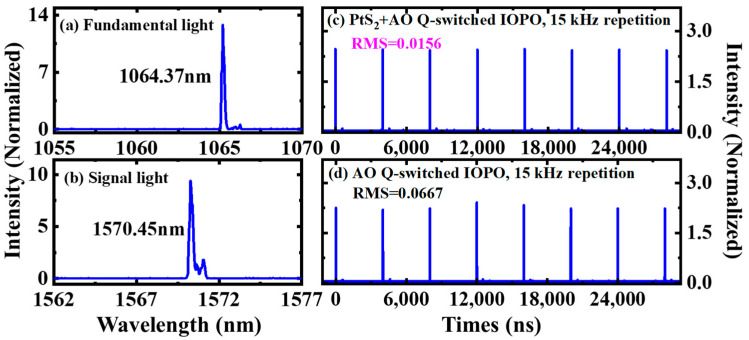
Spectrum and temporal pulse train. (**a**,**b**), corresponding spectrum from active–passive Q-switched IOPO at incident pump of 5.0 W and *f_p_* = 15 kHz. (**c**,**d**), typical temporal pulse train of signal light from Q-switched IOPO, under incident pumped power of 5.2 W and *f_p_* = 15 kHz. RMS: root means square error.

The output spectrum of doubly Q-switched IOPO is shown in Figure 4a,b. The fundamental and signal light wavelengths are found to locate at 1064 nm and 1570 nm, respectively.

Figure 4c,d display the typical pulse trains of signal light from two kinds of IOPO, under the AOM repetition rate of 15 kHz and incident pumped power of 5.0 W. The root means square error (RMS) of peak-to-peak jitter for signal light output from the doubly Q-switched IOPO is only 0.0156 value was much smaller than the 0.0667 RMS of AO singly Q-switched IOPO. As shown in Figure 4, it was concluded that the application of PtS_2_ SA effectively improves the peak-to-peak pulse stability of Q-switched IOPO.

**Figure 5:**
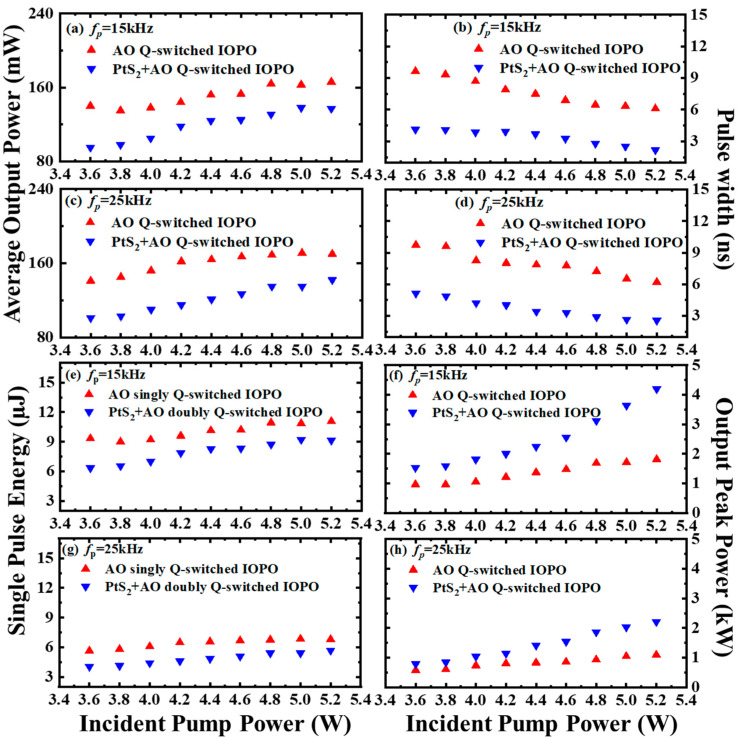
Output characteristics from two kinds of Q-switched IOPO. (**a**,**c**), average output power. (**b**,**d**), pulse width. (**e**,**g**), single pulse energy. (**f**,**h**), peak power.

The laser characteristics of AO active Q-switched IOPO and PtS_2_ + AO dual-loss modulated Q-switched IOPO at two different AO modulation rates, as shown in Figure 5. Figure 5a,c show the average output powers of these two kinds of modulation versus the incident pump powers. The 1570 nm-wavelength average output power of the singly loss-modulated IOPO was indeed higher than the dual-loss-modulated IOPO. This is attributed to the insert loss of PtS_2_ SA. Compared to 3.0 W threshold of AO Q-switched IOPO, the threshold pump power of the dual-loss Q-switched operation increased to 3.6 W.

The pulse width of signal light with different pulse repetitions is shown in Figure 5b,d, which commonly declines with increasing pump power but increases with a rising AO repetition rate. In a dual-loss Q-switched laser, the number of longitudinal modes could be suppressed by the participation of SA, and then the pulse width could be cut down. Therefore, in Figure 5b,d, the signal-light pulse from IOPO pumped by a PtS_2_ + AO doubly Q-switched laser was shorter than that from IOPO pumped by AO singly Q-switched laser. Because of PtS_2_’s saturable absorption to fundamental laser, the signal-light pulse width is 64.3% compressed from 6.102 ns to 2.178 ns at 15 kHz pulse-repetition rate and 5.2 W pump power.

With the average output power and the repetition frequency of the pulse, the pulse energy of the signal light could be calculated by *E_pulse_* = *P_average_*/*f_p_*, which is shown in Figure 5e,g. Then, the peak power was calculated by *P_peak_* = *E_pulse_*/W, where W is the full width at half maximum (FWHM) of the signal pulse [32], shown in Figure 5f,h. Owing to the pulse compression of PtS_2_ SA, the peak power of dual-loss Q-switched IOPO increased 131% from 1.815 kW in single Q-switched IOPO to 4.193 kW in doubly Q-switched one at 5.2 W pump power.

**Figure 6:**
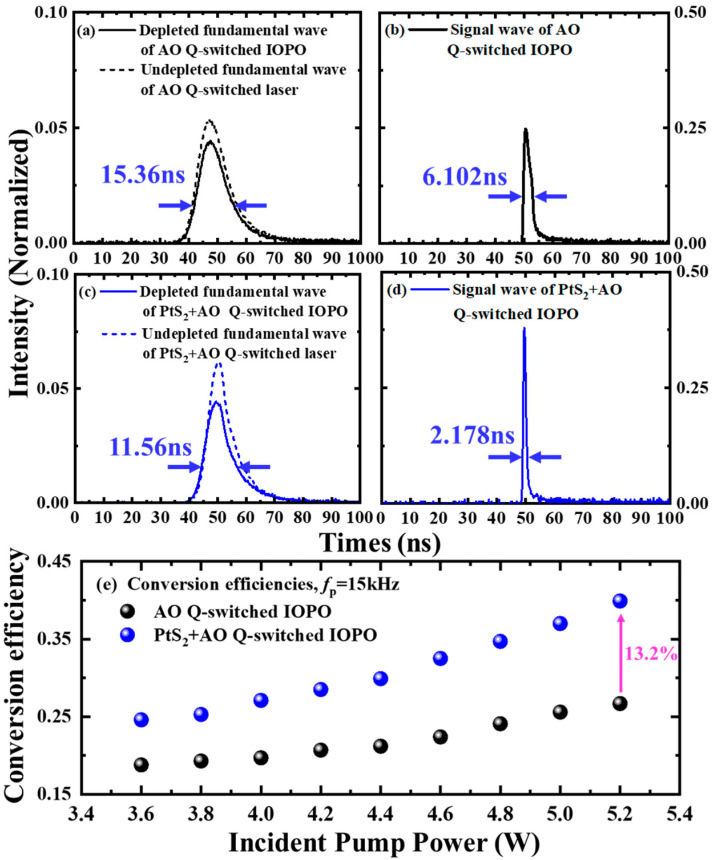
Investigation of IOPO’s conversion efficiency. (**a**,**c**), undepleted and depleted fundamental-pulse profiles from Q-switched laser cavity under incident pumped power of 5.2 W and *f_p_* = 15 kHz. (**b**,**d**), signal-pulse profiles from Q-switched IOPO under incident pump power of 5.2 W and *f_p_ =* 15 kHz. (**e**) fundamental-to-signal conversion efficiencies from two IOPOs versus incident pump power when *f_p_* = 15 kHz.

The temporal pulse profiles of fundamental and signal waves are shown in Figure 6a–d for two IOPOs at the incident pump power of 5.2 W and AO modulation rate of 15 kHz. In Figure 6a, the 1064 nm fundamental-pulse profile leaking from IOPO is shown, and so is the undepleted 1064 nm pulse profile from the AO Q-switched laser without IOPO. For comparison, the depleted and undepleted 1064 nm-pulse temporal profiles from the PtS_2_-based IOPO and laser are shown in Figure 6c. Because of PtS_2_’s saturable absorption, the consumption of fundamental-light energy is larger in doubly Q-switched IOPO than that in AO Q-switched IOPO. In Figure 6e, the fundamental-to-signal conversion efficiency is calculated with the consumption of 1064 nm laser demonstrated in Figure 6a,c. Under incident pump powers of 5.2 W and *f_p_ =* 15 kHz, the nonlinear conversion efficiency of doubly Q-switched IOPO is 39.9%, which increased 13.2% compared to IOPO without PtS_2_. The energy conversion from 808 nm-pump laser to 1570 nm-signal light is up to 3.29%.

**Figure 7:**
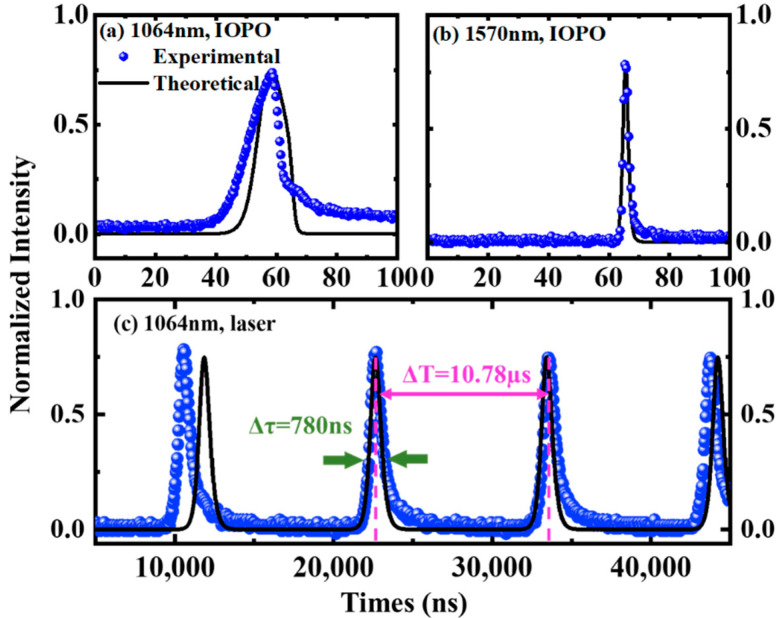
Theoretical fit of temporal-pulse profile output from Q-switched laser or IOPO based on PtS_2_ SA. (**a**) Temporal-pulse profile of the fundamental light. (**b**) Temporal-pulse profile of the signal light. (**c**) Pulses from PtS_2_ SA passively Q-switched laser. Solid, calculated values from rate equations. Scatter, experimental data.

By numerically solving the above-derived rate Equations (4)–(8), the temporal pulse from IOPO or laser could be calculated with PtS_2_’s SA parameters in Table 1. Figure 7a,b show the theoretical and experimental pulse profiles of the fundamental light and signal light at 5.2 W pump power and 15 kHz AO-modulating rate. Figure 7c provides the calculated 1064 nm-wavelength pulse train of the PtS_2_ singly Q-switched laser. It can be seen that the theoretical values from Gaussian rate equations fit the experimental data well.

Several SAs have been used to improve the performance of IOPO. In Table 2, the output characteristics of doubly Q-switched IOPO with AOM and different SA are reviewed. Compared to other SAs, the 6.2 nm-thickness PtS_2_ used in this work could optimize IOPO’s operation at lower incident pump power (≤5.2 W). Meanwhile, doubly Q-switched IOPO based on PtS_2_ attains 3.6 W, the lowest threshold, and 3.29%, the highest pump-to-signal conversion efficiency.

## 5. Conclusions

In conclusion, a 6.2 nm-thickness PtS_2_ SA was prepared using the method of EBE combined with post-sulfurization. Based on the measured nonlinear transmittance, the ground-state and the excited-state absorption cross-sections of PtS_2_ SA were estimated to be 6.4298 × 10^−19^ cm^−2^ and 2.5927 × 10^−19^ cm^−2^, and the excited-state lifetime was approximately 1.043 ms. In the experiment, a KTP IOPO pumped by an active–passive Q-switched fundamental laser with AOM + PtS_2_ was realized. With the application of PtS_2_ SA, the peak-to-peak stability of Q-switched IOPO improved by 76.6%. The pulse width of signal light was 64.3% compressed, and the peak power improved 131%. In particular, due to PtS_2_-SA’s optimization, the conversion efficiency of IOPO improved by 13.2%. To the best of our knowledge, the 3.29% pump-to-signal conversion efficiency is the highest value to date for the doubly Q-switched IOPO with AOM. In theory, the rate equations under Gaussian assumption are derived and numerically solved with PtS_2_’s SA parameters. The theoretical values fit the experiment ones well.

## Figures and Tables

**Table 1:** The key parameters for saturable absorption properties of 2D-PtS_2_ SA.

Parameters	Values
*σ_g_*	6.4298 × 10^−19^ cm^−2^
*σ_e_*	2.5927 × 10^−19^ cm^−2^
*τ_y_*	1.043 ms
*l_y_*	6.2 nm
*n_y_*_0_	5.64 × 10^24^ cm^−3^

**Table 2:** Performances of KTP-IOPOs pumped by doubly Q-switched lasers with an AOM and different SA.

Q-Switching Method	Nonlinear Medium	Max Pump Power (808 nm)	Threshold Value (1570 nm)	Peak Power (1570 nm)	Conversion Efficiency at Max Pump Power (808→1570 nm)	Ref
AOM + Cr^4+^:YAG	KTP	6.3 W	5.1 W	2.25 kW	1.19%	[32]
AOM + monolayer graphene SA		11.7 W	5.2 W	7.9 kW	0.81%	[36]
AOM + MoS_2_ SA		10.2 W	5.6 W	21.6 kW	1.79%	[37]
AOM + MoSe_2_ SA		7.6 W	6.0 W	3.37 kW	1.46%	[33]
AOM + WS_2_ SA		10.2 W	5.2 W	28.7 kW	2.28%	[38]
AOM + PtS_2_ SA		5.2 W	3.6 W	4.193 kW	3.29%	This work

## Data Availability

Not applicable.
